# Supplementary material for: Integrating Network Pharmacology and Transcriptomic Strategies to Explore the Pharmacological Mechanism of Hydroxysafflor Yellow A in Delaying Liver Aging
Source: Int J Mol Sci. 2022 Nov 18;23(22):14281. doi: 10.3390/ijms232214281 (PMC9697017; doi:10.3390/ijms232214281)
Supplement: Supplementary file 1 [file ijms-23-14281-s001.zip › Supplementary material.pdf]

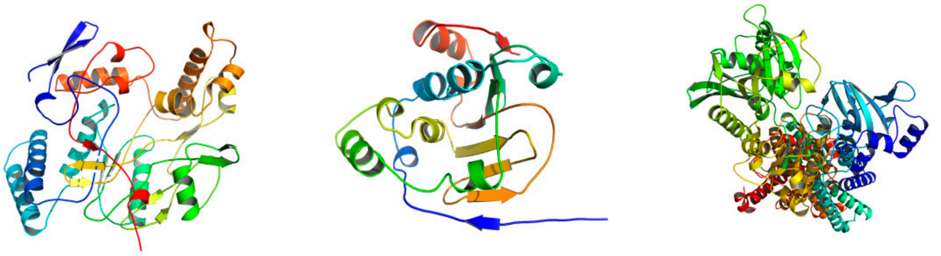

NOS1	HSP90AA1	ATP2A1
**Center_x = 120.75**	**Center_x = 4.67**	**Center_x = -14.95**
**Center_y = 249.22**	**Center_y = 2.05**	**Center_y = -3.31**
**Center_z = 358.56**	**Center_z = 7.89**	**Center_z = 11.38**

Figure S1. Parameters of molecular docking between HSYA and targets.

A

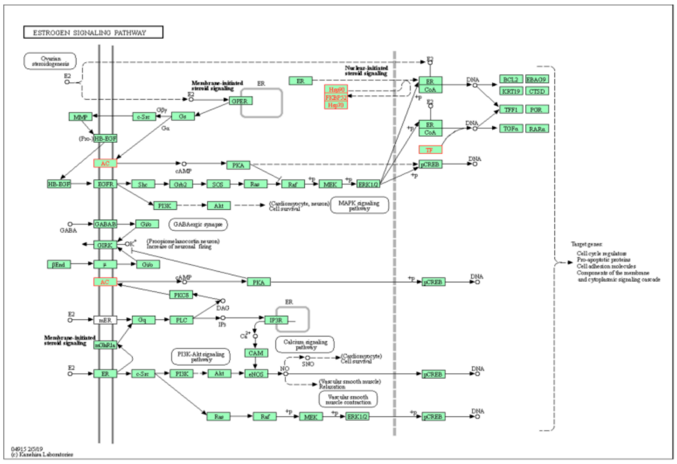

B

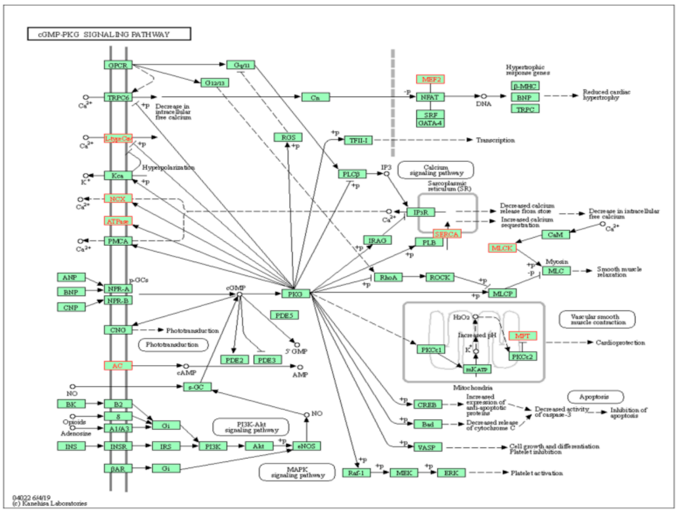

C

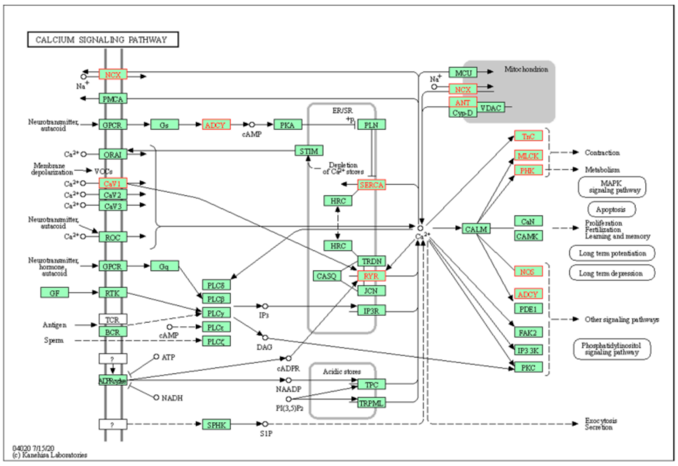

Figure S2. (A) Significantly enriched KEGG pathways in Estrogen signaling pathway. (B) Significantly enriched KEGG pathways in cGMP-PKG signaling pathway. (C) Significantly enriched KEGG pathways in Calcium signaling pathway. The location of action of key targets are marked in red. The pictures were drawn by KEGG Mapper (www.kegg.jp/kegg/tool/map_pathway2.html).

Table S1. Target information of HSYA in delaying liver aging in network pharmacology.

No.	Gene symbol	Protein names	BC	Degree
1	*MAPK1*	mitogen-activated protein kinase 1	0.21348914	29
2	*STAT3*	signal transducer and activator of transcription 3	0.18284102	25
3	*PIK3CA*	phosphatidylinositol-4,5-bisphosphate 3-kinase catalytic subunit alpha	0.1512005	25
4	*HSP90AA1*	heat shock protein 90 alpha family class A member 1	0.07971855	20
5	*EGFR*	epidermal growth factor receptor	0.09310696	18
6	*PLCG1*	phospholipase C gamma 1	0.03314456	17
7	*APP*	amyloid beta precursor protein	0.14783386	17
8	*PRKCD*	protein kinase C delta	0.02400771	15
9	*PRKCA*	protein kinase C alpha	0.02165484	15
10	*IL6*	interleukin 6	0.06431227	14
11	*EP300*	E1A binding protein p300	0.10007231	14
12	*PRKCQ*	protein kinase C theta	0.04664503	13
13	*PRKCB*	protein kinase C beta	0.01164182	13
14	*JUN*	Jun proto-oncogene, AP-1 transcription factor subunit	0.06218907	12
15	*ESR1*	estrogen receptor 1	0.02636277	12
16	*PRKACA*	protein kinase cAMP-activated catalytic subunit alpha	0.07013458	11
17	*MTOR*	mechanistic target of rapamycin kinase	0.0706448	11
18	*IL2*	interleukin 2	0.01018206	11
19	*PTAFR*	platelet activating factor receptor	0.02275635	10
20	*IL1B*	interleukin 1 beta	0.02994886	10
21	*PPP2CA*	protein phosphatase 2 catalytic subunit alpha	0.02972321	9
22	*HCK*	HCK proto-oncogene, Src family tyrosine kinase	0.01419482	9
23	*F2*	coagulation factor II, thrombin	0.0189332	9
24	*VAV1*	vav guanine nucleotide exchange factor 1	0.02422145	8
25	*PGR*	progesterone receptor	0.01262398	8
26	*OPRD1*	opioid receptor delta 1	0.0372198	8
27	*LYZ*	lysozyme	0.02390089	8
28	*HTR1A*	5-hydroxytryptamine receptor 1A	0.01574803	8
29	*HSPA8*	heat shock protein family A (Hsp70) member 8	0.02388195	8
30	*CNR1*	cannabinoid receptor 1	0.01574803	8
31	*ADORA1*	adenosine A1 receptor	0.06149231	8
32	*PLK1*	polo like kinase 1	0.01908619	7
33	*MMP9*	matrix metallopeptidase 9	0.0239186	7
34	*ITPR1*	inositol 1,4,5-trisphosphate receptor type 1	0.00923197	7
35	*ARG1*	arginase 1	0.01062385	7
36	*AR*	androgen receptor	0.00918955	7
37	*PTPN1*	protein tyrosine phosphatase non-receptor type 1	0.12113136	6
38	*MET*	MET proto-oncogene, receptor tyrosine kinase	0.01255273	6
39	*IGF1R*	insulin like growth factor 1 receptor	0.02090308	6

40	*ICAM1*	intercellular adhesion molecule 1	0.01320018	6
41	*GSK3B*	glycogen synthase kinase 3 beta	0.00846765	6
42	*PTGS2*	prostaglandin-endoperoxide synthase 2	0.01629677	5
43	*PPP1CC*	protein phosphatase 1 catalytic subunit gamma	0.00780971	5
44	*NOS2*	nitric oxide synthase 2	0.01861891	5
45	*CDK2*	cyclin dependent kinase 2	0.04431868	5
